# Experimental characterization of speech aerosol dispersion dynamics

**DOI:** 10.1038/s41598-021-83298-7

**Published:** 2021-02-17

**Authors:** Zu Puayen Tan, Lokesh Silwal, Surya P. Bhatt, Vrishank Raghav

**Affiliations:** 1 Department of Aerospace Engineering, Auburn University, Auburn, AL 36849 USA; 2 Department of Mechanical Engineering, National Chiao Tung University, Hsinchu, 30010 Taiwan; 3 University of Alabama at Birmingham, Birmingham, AL 35294 USA

**Keywords:** Respiratory tract diseases, Risk factors

## Abstract

Contact and inhalation of virions-carrying human aerosols represent the primary transmission pathway for airborne diseases including the severe acute respiratory syndrome coronavirus 2 (SARS-CoV-2). Relative to sneezing and coughing, non-symptomatic aerosol-producing activities such as speaking are highly understudied. The dispersions of aerosols from vocalization by a human subject are hereby quantified using high-speed particle image velocimetry. Syllables of different aerosol production rates were tested and compared to coughing. Results indicate aerosol productions and penetrations are not correlated. E.g. ‘ti’ and ‘ma’ have similar production rates but only ‘ti’ penetrated as far as coughs. All cases exhibited a rapidly penetrating “jet phase” followed by a slow “puff phase.” Immediate dilution of aerosols was prevented by vortex ring flow structures that concentrated particles toward the plume-front. A high-fidelity assessment of risks to exposure must account for aerosol production rate, penetration, plume direction and the prevailing air current.

## Introduction

Contact and inhalation of virions-carrying human aerosols represent the primary pathway of transmission for many airborne diseases, including the severe acute respiratory syndrome coronavirus 2 (SARS-CoV-2)^1,2^ of 2020. Among the studies that have been devoted to understand the production and dispersion of human aerosols, most have focused on coughs and sneezes^3–15^, as a mode of disease transmission after the onset of symptoms. In contrast, there is significantly less understanding of non-symptomatic aerosol-producing activities such as speaking. This has become a significant issue during the SARS-CoV-2 pandemic with large proportions of infected persons remaining asymptomatic but infectious during the virus incubation period and/or throughout the course of infection. In fact, a recent study found that pre-symptomatic persons can shed SARS-CoV-2 virus at a higher rate than symptomatic individuals^16^. Exacerbating this issue, asymptomatic persons are also less likely to observe transmission-mitigating measures enacted by health agencies like the United States’ Centers for Disease Control and Prevention (CDC)^17^, thereby increasing their transmission probability. Collectively, these factors highlight the importance of understanding aerosol production and dispersion from everyday activities carried out by asymptomatic persons (“asymptomatic activities”) with regards to mitigating both the COVID-19 and future pandemics. In this study, we focus on the specific asymptomatic activity of speaking, to elucidate the characteristics of speech-generated aerosols, and to determine whether speaking constitutes as much transmission risk as that observed for coughs and sneezes.

Driven by the COVID 19 pandemic, recent research in speech aerosol has gained new momentum^12,18–22^. Studies highlighted that the film burst mechanism responsible for aerosol production in coughs and sneezes is also prevalent in speaking, and is additionally manifested by the vibrations of vocal folds during speech^12^. Furthermore, since speech is carried out over prolonged durations, its cumulative aerosol release may exceed those of coughs and sneezes^23^. Phenomenological, speech-based aerosol production is more complex than coughing and sneezing due to the varied forms of vocalizations, including different consonants, vowels, and durations of speech. In one series of emerging studies, researchers found that certain vowels and consonants consistently exhibit very high rates of aerosol production, while other vocalizations generated low amounts of aerosols^21,22^. The sizes of these aerosol droplets can range from the lower measurement limit of 0.5 µm to approximately ~ 10 µm, peaking at ~ 1 µm^21,22^. This compares against coughs and sneezes that contain both 60–100 µm ballistic droplets as well as small ~ 15 µm aerosol droplets that linger in the ambient air for hours^5,8,9,13–15^.

While the sizes and production rates of speech aerosols have been characterized, their subsequent dispersions have not been investigated; which makes the risk assessment of disease transmission via speech aerosols incomplete. In particular, it is unknown whether speech aerosols reach as far as cough and sneeze aerosols, and by extension as far as CDC’s^17^ 2 m (6 ft) physical distancing guideline. Furthermore, extrapolations of existing cough and sneeze models to estimate the dispersion of speech aerosols is also untenable, due to the wide range of human vocalizations; e.g. different syllables are associated with vastly different mouth shapes and potentially different aerosol ejection velocities, whose influences on the dispersion pattern are also unknown.

Our investigation addresses this gap by experimentally characterizing the dispersion of aerosols produced by a human subject vocalizing several key syllables *and* coughing at different sound intensity levels. The experiments were conducted at Auburn University and was approved by the Institutional Review Board (IRB) under protocol #20-206 EP 2004. Time-resolved planar particle image velocimetry (PIV) was employed to capture the evolution of the generated aerosol-laden gas puffs. A virtual aerosol tracking approach was then overlaid on the PIV data to observe propagation of the otherwise invisibly small aerosol droplets. In this paper the following results are presented: (1) peak aerosol ejection velocity at the subject’s mouth for each test case, (2) evolution of the aerosol plume’s structures, (3) an assessment of whether speech aerosols follow cough’s and sneeze’s classical two-stage dispersion model, and (4) a comparison of penetration potentials for the tested syllables and coughs. With these results, the study seeks to determine whether syllables with the highest aerosol production rates also exhibit the furthest penetration and vice-versa, where the combination of both factors affects transmission risk. The study also determines whether specific zones relative to the speaker are more susceptible to exposure of virus-laden aerosol droplets. And finally, whether speech aerosols penetrate as far as coughs and the CDC’s “6 ft social distancing” guideline.

## Results

Our test matrix follows from Asadi et al.’s studies of aerosol droplet sizes and production rates for different speech syllables^21,22^. They observed that vowels generate more aerosols than consonants. Among the three corners of the International Phonetic Alphabet (IPA) vowel chart^24^, /i/ (as in ‘need’) produced significantly more aerosols than /a/ (‘saw’) or /u/ (‘mood’). The pattern persisted when vowels were connected to a consonant; e.g. ‘heed’ having more droplets than ‘hood.’ On the other hand, for a fixed vowel, consonants belonging to voiced plosive (‘da’, ‘ba’, ‘ga’) and nasal sound (‘ma’, ‘na’) generated the most aerosols, followed by voiceless plosives (‘ta’, ‘pa’, ‘ka’), and then voiced fricatives (‘za’, ‘va’). Voiceless fricatives (‘ha’, ‘fa’, ‘sa’, ‘sha’) produced the least aerosol. Droplets size distributions broadened but maintained a near-constant peak of ~ 1 µm when sound intensity level (SIL) was increased. Aerosol production rates also increased with SIL, as expected.

To determine whether syllables with high aerosol production rates are also associated with high penetrations, this study’s test matrix (Table 1) was designed around the consonants ‘m,’ ‘t’ and ‘s’ in the order of highest to lowest production rates. The consonant ‘t’ was of particular interest to our study because it has a high production rates and also the potential for the strongest aerosol ejection with furthest penetration, due to its stop at the start of vocalization (i.e., the air from vocal tract is fully blocked and then released upon vocalization, resembling an impulsively-started jet). ‘t’ was paired with the vowels /a/ and /i/ with their respective low and high aerosol product rates. Two forms of /a/ pronounced “aw” and “ah” were characterized. Notably, all three vowels have different degrees of mouth opening at the end of vocalization, which will likely affect the characteristics of the ejected aerosol jet. In contrast to ‘t’, ‘m’ does not contain a stop and is expected to produce significantly softer jets. In fact, the vocalization of pure ‘ma’ did not produce aerosol that penetrated our measurement domain; hence, an alternative version where ‘ma’ was reflexively followed by soft exhale was measured instead, resembling ‘mah.’ Three SILs were tested for the case of ‘t,’ ranging from whispering to normal conversation to loud. For comparison, coughs at three different SILs were measured to represent the classical symptomatic aerosol-producing activity. Notably, this study isolated the physics of speech aerosols to single syllables. The physics of plume-plume interactions when syllables are vocalized in succession was not explored.

**Table 1:** Test points.

Category	Word	Sound intensity level (SIL)	Duration, $\Delta t_{vocal}$ (s)
Nasal	‘ma’ (muh)	Loud + exhale	0.22
Fricatives	‘sa’	Loud	0.23
	‘si’	Loud	0.22
Plosives	‘taw’ (taw)	Whisper	0.12
		Normal	0.25
		Loud	0.49
	‘ta’ (tuh)	Whisper	0.16
		Normal	0.22
		Loud	0.24
	‘ti’	Whisper	0.06
		Normal	0.20
		Loud	0.29
Cough	–	Whisper	0.26
		Normal	0.20
		Loud	0.40

Peak sound intensity levels (SIL): Loud: $\overline{SIL} =$ 97.5 dB, $\sigma_{SIL} =$ 4.8 dB, Normal: $\overline{SIL} =$ 85.9 dB, $\sigma_{SIL} =$ 2.5 dB, Whisper: $\overline{SIL} =$ 79.9 dB, $\sigma_{SIL} =$ 8.9 dB

### Aerosol ejection velocities

**Figure 1:**
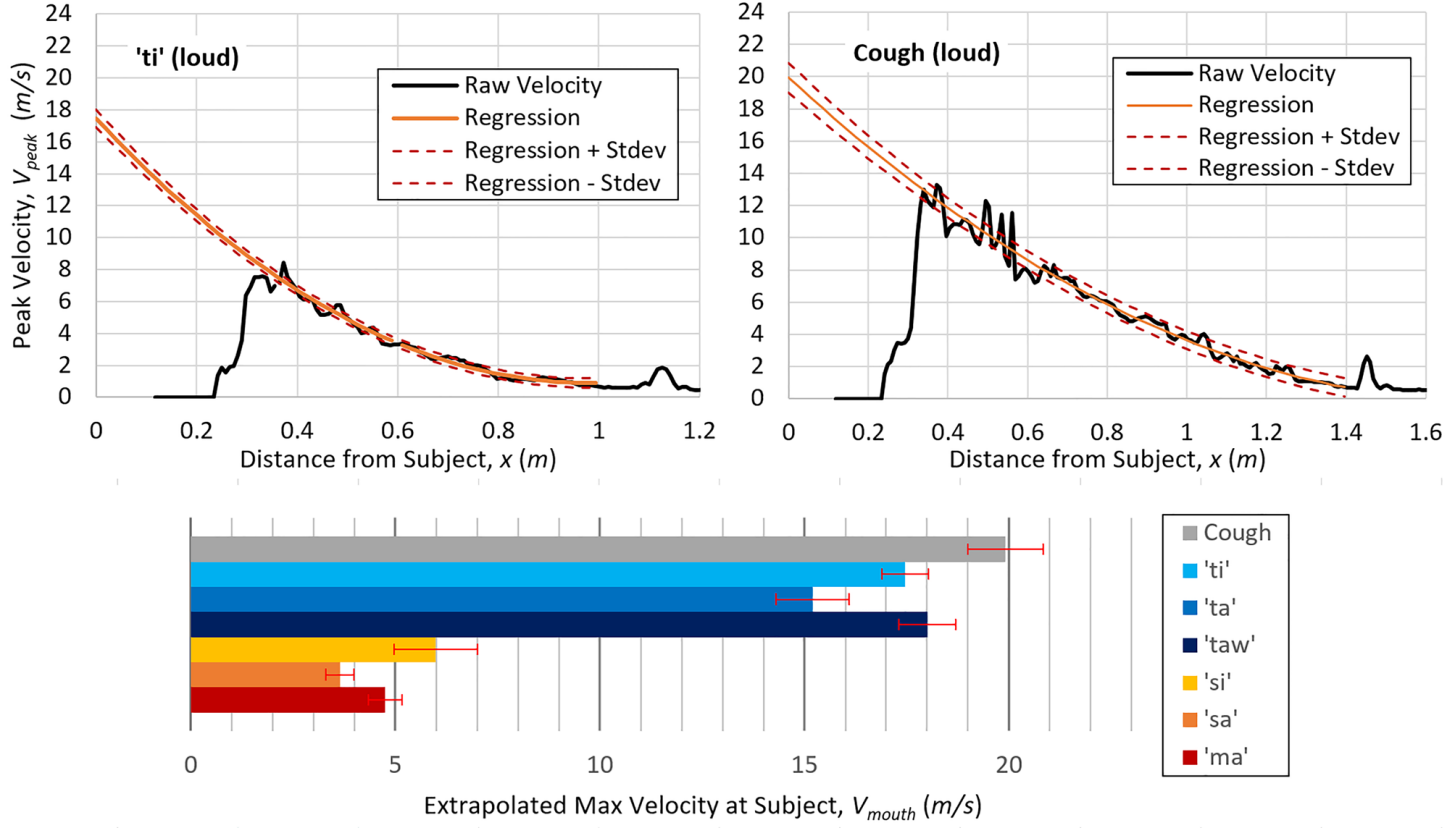
Top: example of velocity extrapolations from loud vocalization of ‘ti’ and cough. Bottom: plots of extrapolated maximum velocity at the subject’s mouth. Error bars correspond to one standard deviation of regression.

Figure 1 summarizes the maximum aerosol ejection velocities at the subject’s mouth ($V_{mouth}$) for vocalizations and cough at loud SIL. Direct PIV measurement at the mouth was not achievable as the laser-sheet had to be aimed away from the subject due to safety protocols. Instead, velocity at the subject’s mouth was determined by extrapolating $V_{peak} \left( x \right)$ from the downstream measurement domain, where $V_{peak} \left( x \right)$ is the local peak velocity at distance $x$ from subject, as encountered during the entire measurement period. Figure 1 shows examples of obtaining $V_{mouth}$ from $V_{peak} \left( x \right)$ for loud ‘ti’ and cough. The aerosol plume’s core was only detectible from 0.4 m forward in both cases, marked by a maximum in the raw $V_{peak}$ curve at this position. Beyond the maximum, $V_{peak}$ decreased quadratically until it merged asymptotically into the background noise levels at approximately 1–1.6 m. Quadratic regression in the form of:

1 $$V_{peak} \left( x \right) = C_{1} x^{2} + C_{2} x + C_{3}$$

where $C_{i}$ are fit coefficients was performed on the region between the maximum and merging with background, and used to extrapolate peak velocity to the subject’s mouth: $V_{peak} \left( {x = 0} \right) = V_{mouth}$. The same trends were observed for all vocalization and cough cases, and thus the same regression model was applied with good fit. Dashed lines on the top of Fig. 1 and error bars on the bottom plot denote one standard deviation of raw data from the regression.

The bottom of Fig. 1 shows the obtained $V_{mouth}$ for tested syllables and cough. Coughing and the plosives cases (‘ti,’ ‘ta,’ ‘taw’) produced significantly higher aerosol-ejection velocities than the nasal and fricative cases (‘ma,’ ‘sa,’ ‘si’). This pattern agrees with the earlier hypothesis that consonants with stops produce faster impulsively started jets. The projected velocity for cough in Fig. 1 agrees with existing literatures^11^ that reported values ranging from 10 to 22 m/s. Especially in the case of ‘t,’ the effect of vowels on $V_{mouth}$ appears marginal. All ‘t’ cases exhibited velocities comparable to coughing, while ‘m’ and ‘s’ were only one-third as fast.

### Aerosol plume evolution

**Figure 2:**
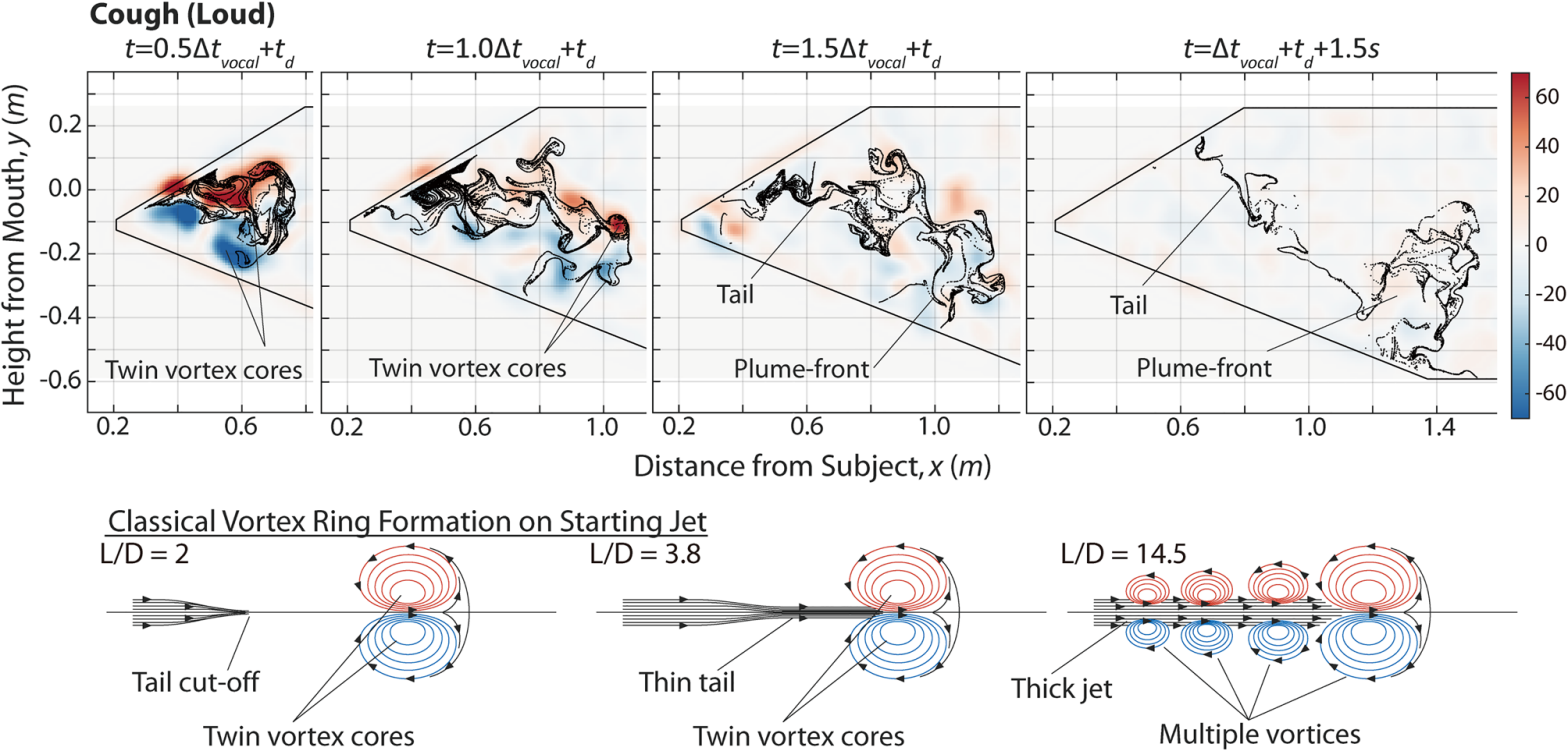
Dispersion dynamics of aerosol particles for a loud cough. Background: vorticity in s^−1^. Inset: illustration of a classical vortex ring formation process from an impulsively-started jet^25^ for comparison.

While $V_{mouth}$ is one measure of the strength of aerosol ejection, other factors such as mouth shape, which affects the cross-sectional profile, angle, hydraulic diameter and turbulence levels of ejected fluid, can all affect the aerosol plume’s structure and penetration. This section examines the evolution of aerosol plumes for the tested cases, starting with Fig. 2 for the classical case of loud coughing. The black particles in this figure represents individual virtual aerosol droplets (see Methods for detailed description), while the background scalar shows the out-of-plane vorticity field ($\xi = \frac{\partial v}{{\partial x}} - \frac{\partial u}{{\partial y}}$, where $u,v$ are velocity components along $x,y$, whose origin is centered at the subject’s mouth). Time $t = t_{d}$ denotes the instance that the plume enters our measurement domain, while $\Delta t_{vocal}$ denotes the total duration of the subject’s vocalization as per Table 1. Figure 2 shows the aerosol and vorticity distributions at mid-cough ($+ 0.5\Delta t_{vocal}$), at end of cough ($+ 1.0\Delta t_{vocal}$), and at $+ 1.5\Delta t_{vocal}$ to illustrate how the aerosol plume evolved initially. Thereafter, the instance 1.5 s after the cough ended is also shown to illustrate the plume’s long-term behavior. It will be demonstrated later that the aerosol plumes’ jetting-momentum dissipate very rapidly (often before the vocalization is over) and 1.5 s is sufficient to represent plume dispersion dynamics primarily driven by the prevailing background air currents. Notably, since aerosols can only be produced when vocalization is in process, virtual aerosol particles were only released in Fig. 2 for the duration of $\Delta t_{vocal}$ from $t = t_{d}$ onwards. This provides a more physically accurate depiction of aerosol dispersion pattern compared to continuous release.

Coughs and sneezes are often modelled as impulsively started jets. For comparison the bottom of Fig. 2 illustrates the classical structures of such jets^25^. Dependent upon the stroke ratio $L/D$ where $L$ is the length of ejected fluid and $D$ is ejection diameter, three different structures can form, all of them dominated by a large vortex ring leading the plume (illustrated as a twin vortex core in this cross-sectional cut). At $L/D < 4$, the jet is a short puff, and all momentum are absorbed into the vortex ring. When $L/D \approx 4$, the maximum amount of momentum has been absorbed and the vortex ring begins to trail a thin tail. At $L/D > 4$, the vortex ring is unstable and sheds off smaller vortices. The strong jet also continues to push forward in a thick column.

An order of magnitude estimation based on $V_{mouth}$, $\Delta t_{vocal}$ and approximate mouth diameter $D$ of 50 mm places loud cough’s $L/D = V_{mouth} \Delta t_{vocal} /D$ at $\sim 160$. Thus, a loud cough contains far more jetting momentum than a single vortex ring could entrain. Figure 2 shows that the cough plume at $+ 0.5\Delta t_{vocal}$ exhibited a dominant vortex ring along the plume-front at first, similar in structure to the classical impulsively start jet. But as expected of $L/D\sim 160$, the vortex ring quickly disintegrated into multiple smaller vortex cores starting at $+ 1.0\Delta t_{vocal}$. At $+ 1.5\Delta t_{vocal}$, the original vortex ring structure has become indistinguishable. However, most ejected aerosol particles continued to move in close unison even at $+ 1.5 s$ after cough, while trailing a tail of particles that is representative of high $L/D$ impulseively-started jets. These observations suggest virus-laden aerosol particles ejected during coughs tend to remain concentrated within a moving plume-front instead of diluting uniformly. Regions where the plume swept through likely contain very low virus concentrations and pose relatively low risk of transmission. In contrast, direct collision with the traveling plume-front is expected to result in very high virus exposure.

**Figure 3:**
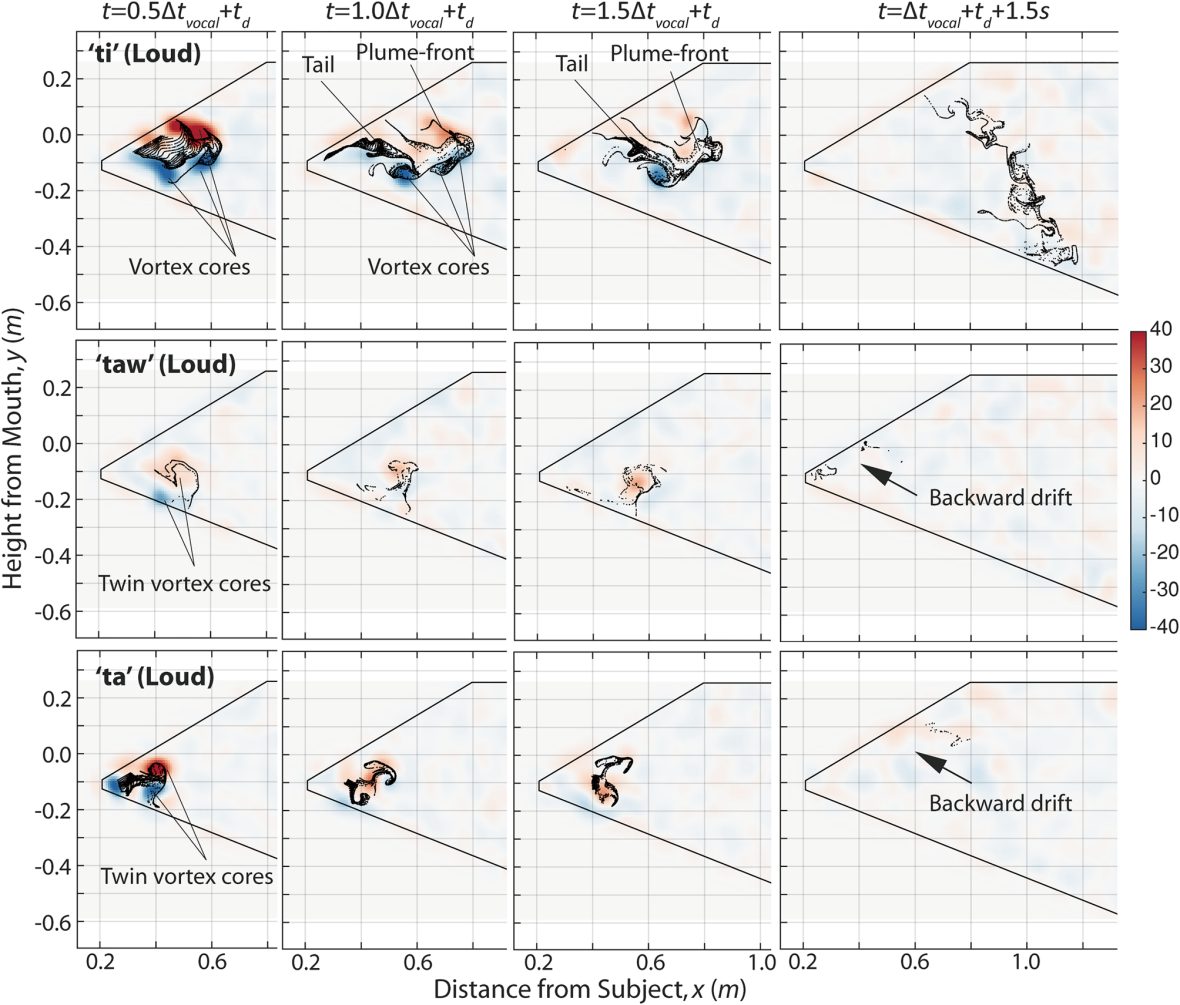
Dispersion dynamics of aerosol particles for plosives. Background: vorticity in s^−1^.

Aerosol dynamics for the voiceless plosive ‘t’ are shown in Fig. 3. Similar to cough, these cases are estimated to have $L/D$ on the order of 100, far exceeding the critical $L/D$ of 4. Despite similar $V_{mouth}$’s across the three plosives (Fig. 1), ‘ti’ produced a plume that penetrated significantly further than both ‘taw’ and ‘ta.’ ‘Taw’ and ‘ta’ both lost their penetration velocity rapidly and travelled less than 0.2 m between $+ 0.5$ and $+ 1.5\Delta t_{vocal}$. By 1.5 s after vocalization, the plumes from ‘taw’ and ‘ta’ were transported backward and upward by the background flow out of the domain of measurement. In contrast, the plume from ‘ti’ continued to travel forward to the 1.2 m mark, comparable with the cough plume that crossed 1.5 m at 1.5 s. Vortex evolutions for ‘taw’ and ‘ta’ are difficult to visualize due to their very short penetration. However, a vortex ring could briefly be observed for both cases as illustrated. In the case of ‘ti’, large swaths of positive vorticity were observed on the top half of the plume and negative vorticity along the bottom half, consistent with a classical vortex ring structure. As expected for its high $L/D$, the vortex structure was unstable and quickly disintegrated. A plume-front and thin tail were vaguely recognizable for ‘ti’ at $+ 1.5\Delta t_{vocal}$, after which the tail was observed to drift upwards while the plume-front was torn and stretched downward. Due to the asymmetric tearing of the plume-front, aerosol particles were dispersed across a large range of distance in ‘ti.’

**Figure 4:**
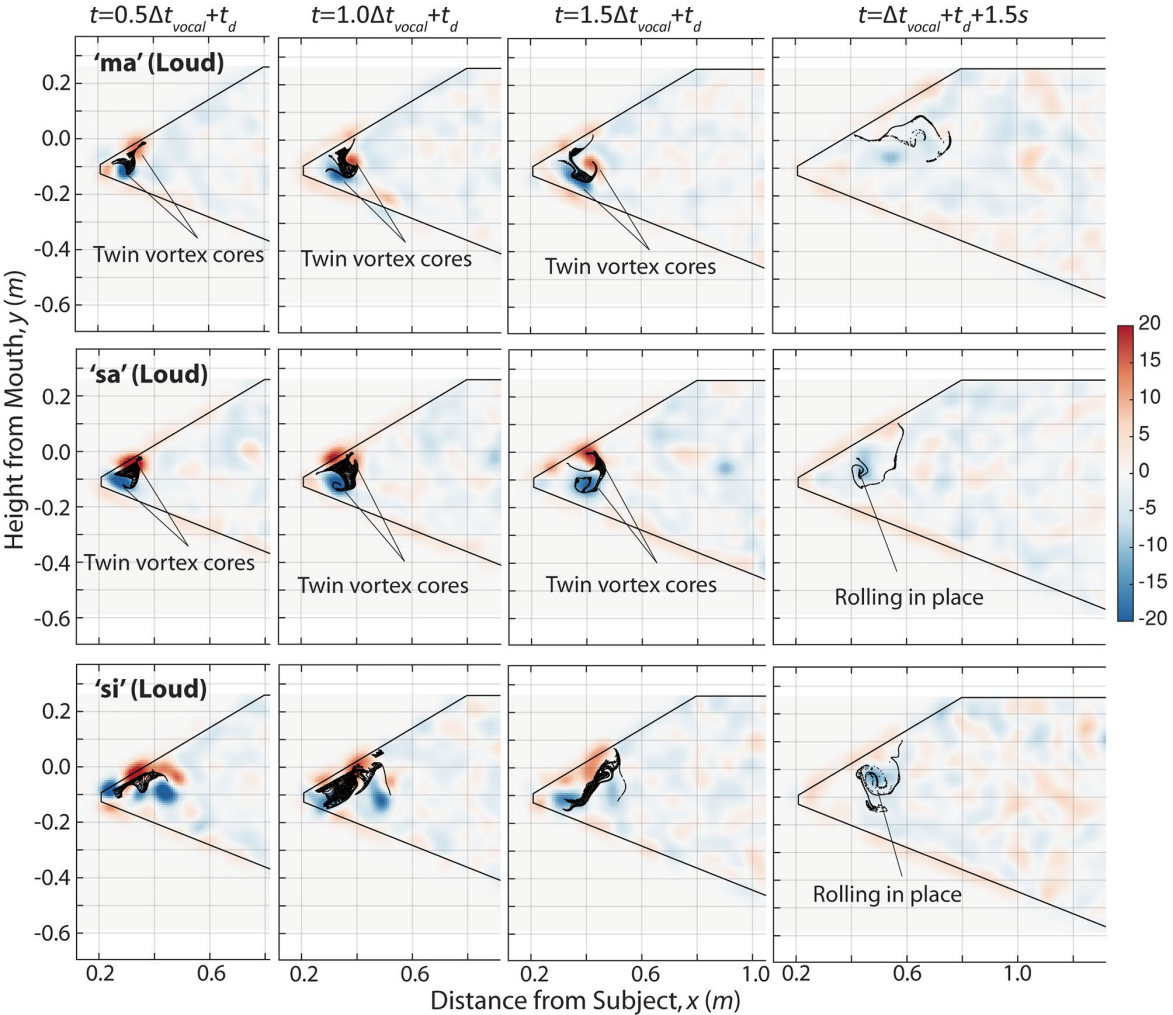
Dispersion dynamics of aerosol particles for nasal and fricatives. Background: vorticity in s^−1^.

Aerosol plumes produced by the nasal sound ‘ma’ and fricatives ‘sa’ and ‘si’ are shown in Fig. 4. In contrast to cough and plosives, their $L/D$ values based on maximum velocity were all on the order of 20, close to the critical $L/D$ for classical starting jets. Due to their low penetration, the leading vortex cores were only briefly observed for ‘ma’ and ‘sa’ but not ‘si.’ In the former two cases, the plumes appear to retain a coherent vortex ring for longer durations (up to $+ 1.5\Delta t_{vocal}$) than cough and plosives, consistent with starting jets having lower $L/D$. Though no identifiable vortex cores remain at 1.5 s after vocalization, all three cases in Fig. 4 still exhibit a coherent swirling plume-front of aerosol particles at this stage, as opposed to cough and plosives where the plume-front became unrecognizably distorted.

### Two-stage transport of aerosol

Numerous fluid mechanics models have been proposed to conceptualize aerosol dispersion in coughs and sneezes, notable among which is the two-stage jet-puff model^7,8,10,15^, wherein the aerosols experience rapid initial penetrations driven by the expired air’s jetting momentum (“jetting phase”). As the jetting momentum dissipates, usually within 0.1 s, the aerosol droplets continue to be slowly transported over an extended period by the plume’s remnant momentum while undergoing additional entrainment mixing with ambient air (“puff phase”). In the latter, aerosol dispersion can be significantly affected by ambient air currents, the expelled gas’ thermal buoyancy and both Brownian and turbulent eddy diffusion. In the first phase, plume-front penetration typically scales as $t^{1/2}$, whereas in the latter a $t^{1/4}$ scaling is typically observed^7,10^. We seek to assess whether the two-stage behavior also manifested within our cough and vocalization plumes.

**Figure 5:**
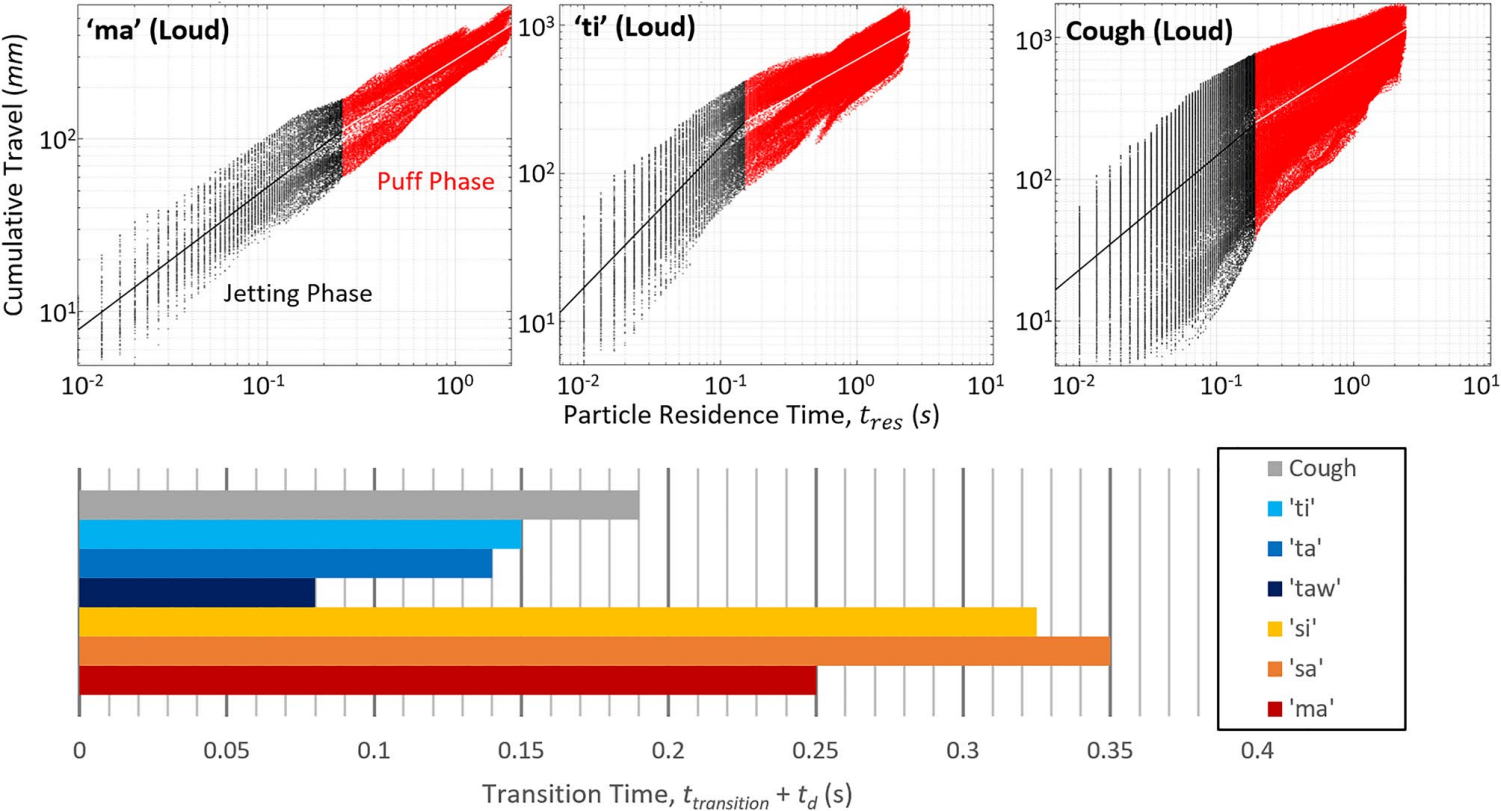
Time-cumulative displacement tracks of aerosol particles for loud vocalizations and cough.

From Figs. 2, 3 and 4, it was evident that the aerosol plumes contained significant structural distortions, including disintegration of the plume-front and up/downward bending of the plume trajectory, as compared to a theoretical jet/puff that remains symmetric and straight in trajectory. Hence, maximum penetration as a function of time could not correctly capture the dynamics of our plumes. Instead, the two-stage behavior was sought through the “dispersion histories” of the virtual aerosol particles, defined as each particle’s cumulative travel distance as a function of residence time since release. As shown in Fig. 5, on a log–log plot of the cumulative travel distance versus residence time, the particles’ dispersion histories manifested the distinct two-stage behavior: a steep linear rise in the first phase that suggests constant-power scaling with time, followed by transition into the second phase with a shallower line that suggests a lower-power scaling. A line has been fitted to each phase and the intersection of these lines defined as the transition time. Similar trends were observed across all cases, suggesting the fundamental two-stage model holds.

The bottom of Fig. 5 shows observed transition time for cough and all cases of vocalization. Interestingly, transition times for cough and the plosive sounds are closer to the 0.1 s found in literature, whereas transition times for the nasal and fricative cases are distinctly longer. This may be due to the cough and plosive cases’ very high $L/D$ compared to nasal and fricative cases’ near-critical $L/D$-or in other words, phenomenologically different regimes of impulsively-started jets.

### Aerosol penetration

**Figure 6:**
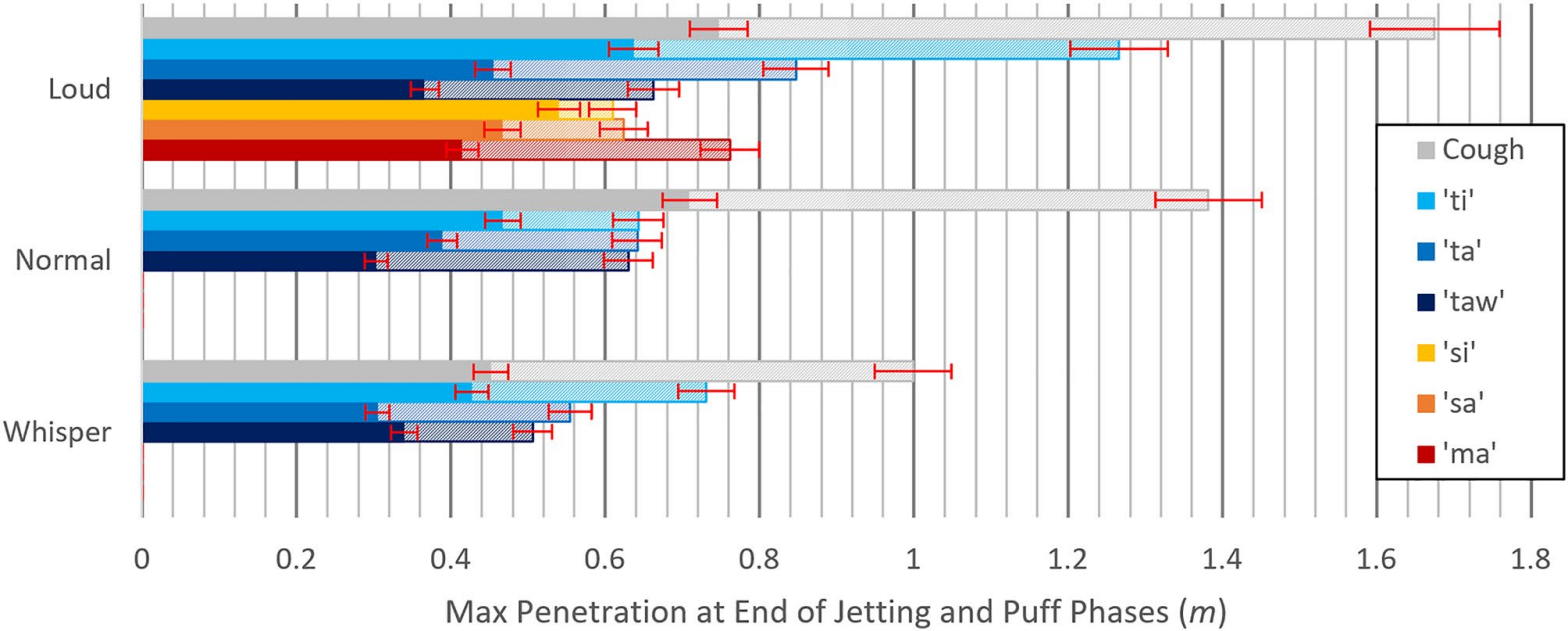
Maximum aerosol penetrations at the end of the jetting phase (filled bars) and subsequent puff phase (empty bars). Uncertainty is estimated based on the velocity measurement uncertainty of the PIV cross-correlation.

Finally, an important goal of this study was to characterize the penetration potential of aerosols generated by different types of vocalizations in comparison to cough aerosol. In this regards, two separate penetration values were derived: (1) maximum penetrations at the end of the jetting phase and (2) maximum penetrations throughout the measured duration (approximately 2 s) which includes the puff phase where plume and background air interactions may significantly affect dispersion. The transition times in Fig. 5 were used to demarcate the end of jetting phase. Figure 6 shows the maximum penetration of aerosol particles within the measurement domain at the end of the jetting phase and puff phase, respectively. The influence of sound intensity level (SIL) on penetration is also shown in Fig. 6 for cough and plosives syllables.

In all cases, the aerosols gained further penetration during the puff phase as expected. However, these gains were especially large for the case of loud ‘ti’ and all coughs. Notably, the loud ‘ti’ achieve penetration that was comparable to a normal cough. In all cases, penetration remained under 2 m, suggesting that “6 feet physical-distancing” may be sufficient for the jetting phase and puff phase within the measured duration of 2 s, but is no safeguard against subsequent transport if air current directions are favorable. Interestingly, despite having significantly higher $V_{mouth}$, ‘taw’ and ‘ta’ obtained similar penetrations as ‘ma’, ‘sa’ and ‘si.’ ‘Ti’ consistently obtained larger penetrations relative to ‘taw’ and ‘ta’, especially during the puff phase in loud SIL. This suggests that fluid dynamically ‘ti’ behaves differently from other syllables, perhaps because of combining a stop consonant with the vowel /i/ that is vocalized with closed mouth, which resembles a converging nozzle that accelerates fluids. Except for the potential outlier of normal ‘ti’ having low penetration at the end of puff phase, higher SIL’s generally produced higher penetration as expected.

Figure 6 showed maximum penetrations up to the point where jet momentum in the puff phase has dissipated. Though not explicitly measured under the current study, aerosol will continue to linger in the air and disperse even after the puff phase. In this post-puff period, dispersion will be dominated by several competing effects that include organized convection by ambient air currents either from room ventilation or thermal buoyancy, Brownian diffusion of the aerosol particles, turbulent eddy-driven diffusion, as well as gravity-driven settling of the particles. Notably, contact with aerosol droplets in post-puff phase can take one of two forms: direct interception by the moving plume or indirect contact after aerosol has become fully diluted with room air. In the following analysis, we primarily focus on the former, which has a more fluid dynamic-related origin.

With regards to settling time, as described in the introduction, aerosol droplets from speech range from 0.5 to 10 µm and peaks at 1 µm^21,22^, while cough droplets include 15 µm aerosol and 60–100 µm ballistic droplets^5,8,9,13–15^. Based on these diameters and assuming Stokes’ flow, the settling velocities for cough droplets are on the order of 3 mm/s, while that of speech droplets are 0.03 mm/s. These values are listed in Table 2 for comparison against other post-puff mechanisms. Hence, given the same penetrations, droplets from ‘ti’ or other syllables are expected to linger in the air longer than cough aerosol.

**Table 2:** Comparison of competing dispersion effects in the post-puff phase.

		Droplet sizes (µm)	Settling velocity^*^ (mm/s)	Ambient air current velocity (mm/s)	Brownian diffusivity (mm^2^/s)	Eddy diffusivity (mm^2^/s)
Speech		$0.1$	$2.95 \times 10^{ - 4}$	0.42–18	$6.97 \times 10^{ - 4}$	10^5^–10^6^
		$1.0$	$2.95 \times 10^{ - 2}$		$2.76 \times 10^{ - 5}$	
	Cough	$10$	$2.95$		$2.40 \times 10^{ - 6}$	
		$100$	N/A		N/A	

*Settling velocity calculated assuming Stokes’ flow.

The settling velocity is also an indicator of how easily the aerosol droplets are stirred and convected by background currents. For the analysis of this effect, typical values for indoor air currents are estimated based on the work of Kohanski et al.^26^ and the American Society for Heating, Refrigeration, and Air-Conditioning Engineers (ASHRAE) standards^27^. Neglecting the effect of convection driven by room occupants’ movements and aerosol plume thermal buoyancy (the latter of which would move aerosol upwards instead of towards other persons), indoor air currents are primarily produced by room ventilation. The magnitudes of ventilation are typically ≥ 6 number of air changes per hour (ACPH) for clinic rooms, ≥ 15 for operating room, ≥ 2 for outpatient rooms as required by ASHRAE, and at least 0.35 for residence, shops and restaurants. Forced air ventilation can be a double-edge sword, where more aggressive ventilation prevents the build-up of diluted aerosol but also generates stronger currents that can convect concentrated aerosol plumes between occupants. Using the values of 0.35–15 ACPH, assuming ventilation occurs uniformly through the entire ceiling surface area (highly conservative velocity) and a typical single-storey height of 4.3 m, we can estimate an ambient air current velocity of 0.42 to 18 mm/s (see Table 2). This is higher than the settling velocities of most speech droplets and some cough droplets. Thus, even the lowest levels of ventilation can help suspend speech droplets indefinitely. Notably, a more realistic ventilation layout where air is pumped in via isolated ducts will likely produce much stronger local currents that can push post-puff aerosol plumes between occupants within several seconds.

Aside from jet/puff and ventilation-driven motions that are both directional, we can also consider dispersion by random Brownian and turbulent eddy diffusions. The former has a diffusivity value, $D$, that is expressed in Eqs. (2) and (3):

2 $$D = C_{c} \left( {\frac{kT}{{6\pi \mu r}}} \right)$$

3 $$C_{c} = 1 + \frac{l}{r}\left[ {1.257 + 0.4\exp \left( { - \frac{1.1r}{l}} \right)} \right]$$

where $C_{c}$ is Cunningham’s correction, $k$ is Boltzmann’s constant, $T$ is temperature, $\mu$ is dynamic viscosity, $l$ is the mean free path of air, and $r$ is the aerosol droplet radius. The resulting values are on the order of $10^{ - 3}$ to $10^{ - 6}$ mm^2^/s (listed in Table 2), with speech droplets having an order of magnitude higher diffusivity. Brownian diffusion alone, however, is typically very slow compared to eddy diffusion. The combination of both mechanisms can be described using a drift-flux model commonly found in the studies of indoor air^28^. While exact values for eddy diffusivity depends on turbulence characteristic of the room (which can draw energy from ventilation-generated shear-flow), Gorbunov estimated that they are on the order of $10^{5}$ to $10^{6}$ mm^2^/s for venues such as street canyons^29^-substantially above Brownian diffusivity. Droplet size will play a role in how they follow turbulent eddies, but given sufficiently low particle concentrations, the droplets are not expected to alter turbulence characteristics.

Summarily, we estimate that in the post-puff phase where the initial jet’s momentum has fully dissipated, aerosol particles are expected to linger in the air. Further dispersion is primarily driven by organized ambient air current or, in its absence, by eddy diffusion. Both mechanisms can draw energy from sources of ventilation in the room. Speech droplets, being an order of magnitude smaller than cough droplets, are especially susceptible to these dispersion mechanisms.

## Discussion

Our study showed that coughing and consonants with a stop such as the plosive ‘t’ produced significantly faster starting jets ($V_{mouth}$) than consonants such as ‘m’ and ‘s.’ Vowels have a significantly smaller impact on $V_{mouth}$. The classical vortex ring formation process arising from impulsively-started jets^25^ was manifested in most tested cases. In cough and plosives, the vortex ring structures were unstable due to $L/D$ values on the order of ~ 100. Whereas for nasal and fricative cases the $L/D$ of ~ 20 allowed coherent vortex structure to persist for longer durations. Our results also suggest that virus-laden aerosol particles do not dilute uniformly upon expulsion. Instead, the vortex ring structure caused particles to roll up and concentrate towards the plume-front. The concentration of aerosol particles typically persists and moves in unison even after the underlying vortex has dissipated. In some high $L/D$ cases, a tail of aerosol particles lingered where plume-front swept through.

A two-stage behavior was observed for all cases, though some were more pronounced than others. Strong, high $L/D$ jets such as ‘ti’, ‘ta’, ‘taw’ and cough transitioned more quickly between phases relative to weaker, low $L/D$ jets such as ‘ma’, ‘si’ and ‘sa.’ Consequently, aerosol penetrations by the end of the jetting phase were comparable between all vocalization cases despite very different $V_{mouth}$. However, cough and ‘ti’ attained significantly higher penetrations in the subsequent puff phase. Thus, our results revealed that aerosol production rates and subsequent dispersions are independent processes: the highest aerosol-producing consonants such as ‘t’ and ‘m’ can produce very different penetrations. The combination of production rates and dispersions, which defines virus concentration and swept area, is required to accurately assess the actual risk of transmission by vocalization.

In the context of CDC’s recommended physical distancing, none of the cases’ aerosol plume reached 2 m by the end of the jetting phase or by the end of the measurement duration (2–2.5 s), though their observed susceptibility to transport by prevailing background flow suggests 2 m can be easily exceeded in favorable conditions. Notably, our method of measurement focused on aerosol particles and neglected ballistic droplets that may exceed 2 m. Therefore, the 2 m physical-distancing guideline should be interpreted as allowing time for individuals to move out of an aerosol plume’s vicinity, but not guaranteed against subsequent interception by the plume after more than a few seconds, nor against interception by ballistic droplets. Critically, the study suggests interception by an aerosol plume is very detrimental, since a very large fraction of the ejected aerosols will remain confined within the plume-front instead of diluting. In this regard, speaking may represent a higher transmission risk than coughs and sneezes. The latter are singular events with a plume-front that pass by quickly, whereas the former is a prolonged activity continuously producing plumes of aerosols. Additionally, it is also important to note that the current studies are carried out without the presence of mask which is an important measure for mitigating the spread of aerosol-based airborne disease. More studies are required to characterize speech aerosol in presence of masking^30,31^, as well as in the presence of plume-plume interactions for sequentially vocalized phrases. Finally, an order of magnitude analysis for the post-puff phase suggests that although strong ventilation is a common strategy for reducing aerosol accumulation in a room, it can also become a driver for ambient air currents and eddy diffusion, which accelerates the dispersion of aerosol plume between persons. Ventilation strategies to curtail the spread of virus must thus consider the room’s air flow pattern in relation to occupants.

## Methods

### Human subject

Our findings were generated by evaluation of a single healthy male subject of 34 years of age and 1.77 m in height. Although this limits the statistical scope of the measurements, it is in line with prior studies^21^ that have reported minimal variance by age, gender and BMI in aerosol generation during speech, and in line with recent single-subject studies^32–34^ driven by the need for timely response to COVID-19.

Uncertainties in this study are expected to be mitigated through repeatability of trends for plosives and coughs at different SIL’s, as well as the consistency in penetration and velocity differences between the plosives/coughs group and nasal/fricatives group. The results should be interpreted as a characterization of relative differences between different vocalizations and cough within a single individual, rather than guidance towards average penetration values throughout the population.

The study with human subject was approved by Auburn University Institutional Review Board (IRB) under protocol #20-206 EP 2004. All methods were carried out in accordance with relevant guidelines and regulations. Informed consent was obtained from the human subject for the study.

### Experiment setup

**Figure 7:**
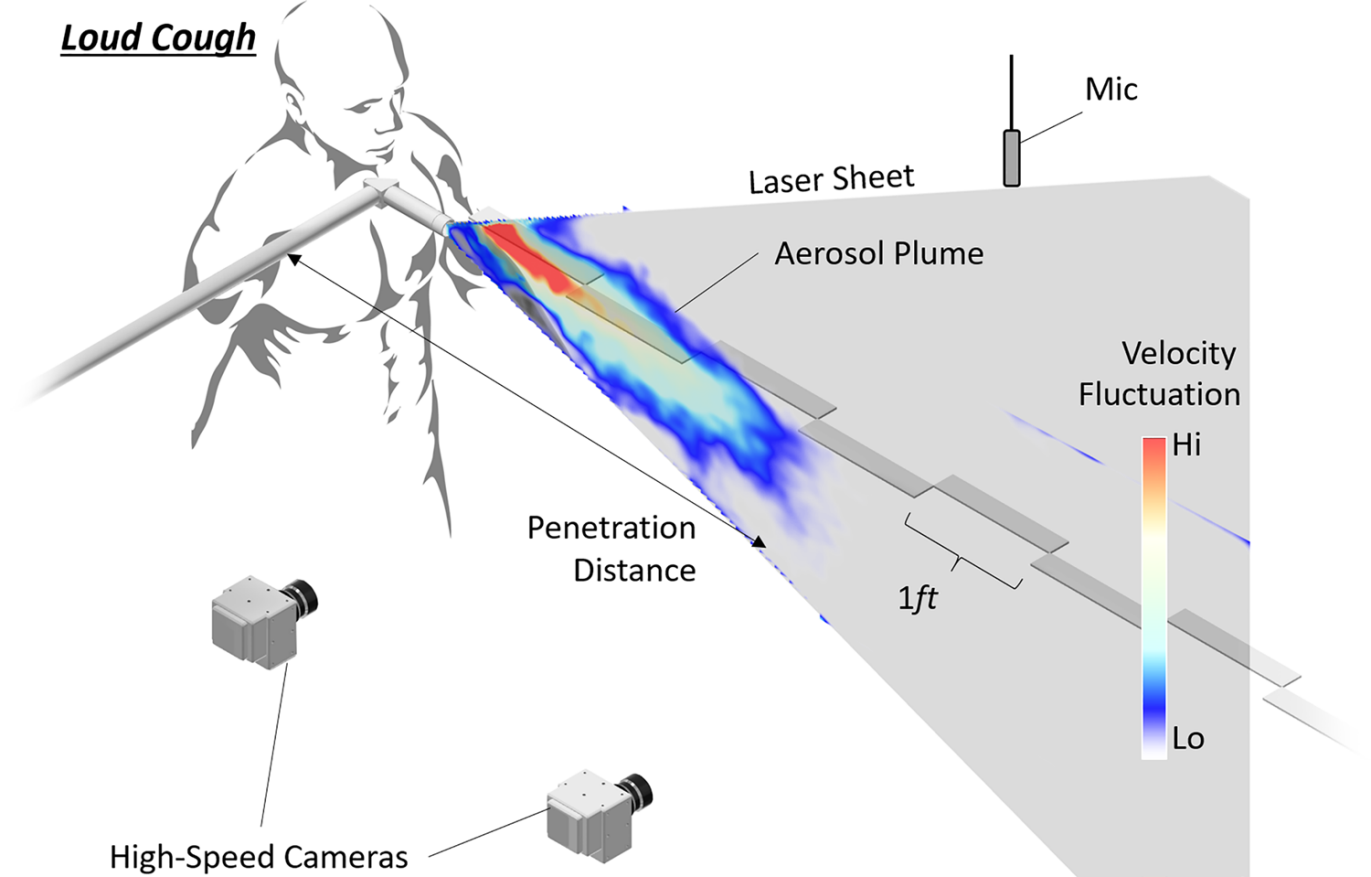
Illustration of the experimental setup superimposed with the velocity of the cough plume.

The experimental setup for this study is illustrated in Fig. 7. The standing subject was positioned in a 42 m^2^ office space, with 1 m of empty space behind, 3 m to the front and at least 1.5 m to the side, sufficient to exclude any aerosol-wall interactions. Penetration distance $x$ was defined as forward from the subject and height $y$ upwards, with the coordinate system’s origin aligned at the subject’s mouth. Background air currents in the room was measured at 0.2 m/s.

The planar measurement domain was aligned to the subject’s mouth and mid-sagittal plane. This domain extended 2.4 m in front of the subject to encompass the 2 m recommended under most common social-distancing guidelines. A standard PIV technique^35^ was used to measure the distribution of flow velocities along this 2D domain. The PIV setup consisted of three parts: flow-seeding particles to visualize motion of the air/aerosol gas, a laser-sheet to illuminate those particles along the domain and cameras to image the illuminated particles.

200 µm neutrally buoyant helium-filled soap-bubbles (HFSB) generated by a LaVision HFSB Generator were used as the flow tracker of choice, which provided strong scattering signals at the 2.4 m scale of measurement. Naturally occurring aerosol droplets from the subject did not elicit sufficient light-scattering to be imaged at this scale. The entire room was uniformly seeded with HFSB prior to and during each vocalization/cough. These HFSB were illuminated using a Photonics dual-head Nd:YLF pulsed-laser (527 nm, 18 mJ/pulse at 300 Hz) which had its beam expanded into a fanning laser-sheet using a combination of articulated laser arm and cylindrical lens. For safety reasons and due to physical constraints of the laser optics, the laser-sheet was aligned pointing away from the subject, with the origin of fanning underneath and approximately 0.2 m in front of the subject’s mouth. This prevented velocimetry measurements directly at the subject’s mouth, which had to be mitigated through backward extrapolation from downstream velocity measurements (Fig. 1).

Two Vision Research Phantom VEO640 high-speed cameras (4MP) recorded particle motions along the laser-sheet. The cameras were arranged side-by-side with slight overlap to achieve a high effective image resolution of approximately 1.9 px/mm for all cases. In combination, the HFSB seeding density and image resolution allowed for vector spacing of 8 mm. The cameras and both heads of laser were synchronized to operate in straddle-mode ($\Delta t$ = 1 ms) with pairs of images taken at 300 Hz, which was empirically found to provide acceptable time-resolved velocity data across the full test range.

To record SIL, a PreSonus microphone (sensitivity-14 mV/Pa) was positioned 1 m in front of the subject (to one side of the laser-sheet). SIL was calibrated to the dB-scale using a Reed R8090 two-level sound calibrator. Background sound levels in the room were recorded immediately after the experiment and subtracted from vocalization/cough data. Reported SIL value for each case was then defined as the peak dB level in the duration of each vocalization/expiratory event.

### Velocimetry data processing and virtual aerosol tracking

A commercially available software (LaVision Inc’s DaVis 10) was used to process the raw camera data. A board spanning the domain of measurement with dots of 20 mm diameter regularly spaced at 60 mm was used to calibrate the camera, which includes establishing the scale of measurement, de-warping the images and stitching measurements from both cameras. A standard set of procedures for PIV data processing was employed, including image background-subtraction, followed by multi-pass PIV calculations (parameters automatically optimized in DaVis given the maximum possible velocity of the loud cough case), and post-processed with 5-frame median-filter and local 2nd-order polynomial fit to smooth the velocity vectors.

Notably, the PIV approach characterized the motion of all flows within the domain of measurement without distinguishing between aerosol and background air. Consequently, a “virtual aerosol tracking” post-processing technique was performed on the PIV velocity results to elucidate aerosol dynamics. The technique consists of 6 steps as follow:

1. An imaginary rake line was positioned along the top-left of the domain, encompassing the edge where the subject’s aerosol could enter.
2. 1000 virtual aerosol particles were instantaneously released at random positions along the rake. Release began at the time-step where changes in velocities are first detected corresponding to arrival of the aerosol plume.
3. The particles’ positions were integrated forward in time by one time-step $\Delta t = \frac{1}{{300\,{\text{Hz}}}}$ based on the underlying PIV velocity-field.
4. Velocity-field from the next time-step was loaded and the particles were integrated again to their next position.
5. 1000 more particles were randomly released along the rake in this new time-step, representing freshly arrived particles from the aerosol plume. Particle release continued for the duration of the vocalization/cough, $\Delta t_{vocal}$, as measured from the microphone data (see Table 1). This implicitly assumed a uniform rate of particle production during vocalization/cough-an assumption that remains to be studied in detail in future investigations.
6. Positions of the existing particles continue to be integrated forward until the end of the measured duration (2–2.5 s).

1000 particles per time-step was determined to be adequate through incremental increase in particle count until convergence was reached. Furthermore, particles that remained static along the rake after release were deemed badly positioned (i.e., particles outside the aerosol plume that should not physically exist) and removed from calculation.

## Data Availability

The datasets generated during and/or analysed during the current study are not publicly available due to the use of human subject but are available in derived form from the corresponding authors upon reasonable request.
